# Gait biofeedback training in people with Parkinson’s disease: a pilot study

**DOI:** 10.1186/s12984-022-01051-1

**Published:** 2022-07-16

**Authors:** Kate McMaster, Michael H. Cole, Daniel Chalkley, Mark W. Creaby

**Affiliations:** 1 School of Behavioural and Health Sciences, Australian Catholic University, Banyo, Brisbane, QLD Australia; 2 Healthy Brain and Mind Research Centre, Australian Catholic University, Melbourne, Australia

**Keywords:** Idiopathic Parkinson disease, Biofeedback, Gait, Biomechanics

## Abstract

**Background:**

People with Parkinson’s disease (PD) are at a high risk of falls, with ~ 60% experiencing a fall each year. Greater mediolateral head and pelvis motion during gait are known to increase the risk of falling in PD, however the ability to modify these aspects of gait has not been examined. Thus, this study aimed to examine whether mediolateral trunk, head and pelvis motion during walking could be successfully decreased in people with PD using real-time biofeedback.

**Methods:**

Participants were provided with real-time biofeedback regarding their mediolateral trunk lean via a visual projection whilst walking along an 8-m indoor walkway. Using the feedback provided, they were asked to reduce the magnitude of their mediolateral trunk lean. Gait was recorded for four conditions (i) Baseline, (ii) Intervention, (iii) immediately Post-Intervention, and (iv) 1-week Follow-Up. Biomechanical variables associated with falls risk were compared between conditions, including normalised mediolateral motion, gait velocity and stride length.

**Results:**

A reduction in mediolateral trunk lean, step length and gait velocity from Baseline to the Intervention and Post-intervention conditions was observed. Contrary to this, increased normalised ML pelvis and trunk motion was observed between the Baseline and Intervention conditions, but returned to Baseline levels in the Post-Intervention condition.

**Conclusions:**

Results from the current study suggest that real-time visual biofeedback may be effective at modifying specific gait characteristics that are associated with falls in PD. Further research is required to better understand the influence of this intervention approach on falls incidence.

*Trial registration* Australian New Zealand Clinical Trials Registry ACTRN12620000994987. Registered 10 June 2020 - Retrospectively registered, https://anzctr.org.au/Trial/Registration/TrialReview.aspx?id=380324

**Supplementary Information:**

The online version contains supplementary material available at 10.1186/s12984-022-01051-1.

## Background

PD is a neurologically degenerative disease that inhibits motor control, inducing bradykinesia, muscle rigidity, akinesia and festination of gait. These symptoms likely contribute to compromised dynamic equilibrium (defined as “control of the body’s center of mass while moving” [1]), and may help explain why ~ 60% of people with PD experience at least one fall a year [2]. Of these falls, about half occur during ambulation [3]. A recent meta-analysis identified a shorter step length and a slower preferred gait velocity as two biomechanical factors that increase an individual’s risk of falling [4]. While some interventions have been able to increase step length and gait velocity, they have not been associated with a reduction in falls incidence [5, 6]. Interestingly, the meta-analyses also identified that, when normalized to gait velocity, greater frontal plane motion of the axial skeleton during walking increases the risk of falls in those with PD [4]. While frontal plane kinematics have been examined during continuous steady state gait in people with PD [7–9], the effects of modifying the frontal plane motion of the axial skeleton on gait mechanics associated with falls risk has not been investigated.

During walking, the motions of the body’s centre of mass (COM) are heavily influenced by the motions of the axial skeleton (head, trunk, and pelvis), which represent ~ 77% of the body’s overall mass [10]. In PD gait, as in healthy gait, the body’s COM oscillates mediolaterally (ML) to help maintain balance during the single leg support phases of gait by placing it closer to the base of support (i.e. the position of the supporting foot). This oscillation is partially achieved by leaning the trunk from side-to-side in the frontal plane. If frontal plane motion of the axial skeleton is excessive however, as observed in PD fallers [4], the COM may move too far laterally relative to the base of support, decreasing dynamic equilibrium and increasing falls risk. Minimizing ML trunk lean may therefore contribute to a reduction in lateral displacement of the COM, facilitating improved dynamic equilibrium. In addition, healthy gait allows the pelvis and trunk to modulate the motion of the head, allowing for more stable visual and vestibular information that may also facilitate falls avoidance. Individuals with PD however often exhibit an ‘en bloc’ motion pattern. This pattern is characterized by a more rigid interaction between the pelvis, trunk and head, with less attenuation from the lower segments (i.e. pelvis) to those higher (i.e. head). Decreasing ML head, trunk or pelvis motion may therefore improve COM control and sensory perception, consequently enhancing dynamic equilibrium and decreasing falls risk.

An emerging body of evidence indicates that subtle changes to walking mechanics can be achieved in a relatively short period of time by utilizing real-time biofeedback [11, 12]. This approach typically involves measuring specific gait mechanics which are then immediately fed-back to the participant in visual, audible or tactile form. Studies in other populations have demonstrated changes in ML trunk lean with as little as a single session of feedback [13, 14]. Whilst there is limited evidence for the use of biofeedback during walking in individuals with PD, similar biofeedback protocols during balance tasks suggest that it has the potential to modify motion patterns in this population [15, 16].

The purpose of this study was therefore to determine (i) the short-term effects of a real-time biofeedback intervention on ML trunk lean in people with PD and (ii) the short-term effects of a real-time biofeedback intervention on other gait parameters associated with falls risk or dynamic equilibrium. We hypothesized that (i) ML trunk lean would decrease as a result of the real-time biofeedback intervention and (ii) other gait parameters would change towards improved dynamic equilibrium and/or reduced falls risk.

## Methods

### Participants

Twenty-four individuals with clinically diagnosed idiopathic PD (18 male and 6 female, 68 ± 7.6 years) participated in this non-randomised laboratory-based intervention study. Participants were a convenience sample recruited from the local community (Brisbane, Australia) between October 2018 and September 2019. Participants were eligible provided they: (a) were diagnosed with PD by a neurologist; (b) presented with PD-related symptoms ranging in severity from 1 to 3 on the Hoehn & Yahr scale; (c) had no significant surgery within the last three months affecting their gait; (d) experienced no recurrent pain or injury affecting their gait; (e) were able to walk without assistance; (f) had no significant visual (Bailey-Lovie high contrast visual acuity < 0.30 logMAR) or cognitive impairment (Mini Mental State Exam (MMSE) score ≥ 24/30); (g) had not received deep brain stimulation; and (h) were aged under 80 years.

All participants provided written informed consent prior to testing in accordance with the Declaration of Helsinki. Data presented here are reported in accordance with the STROBE guidelines and were collected at the Australian Catholic University, Brisbane as approved by the institution’s Research Ethics Committee (Ref: 2018-196 H). Sample size was estimated using G-Power [17] based on a Repeated Measures ANOVA study design, across four time points. As no previous data were available regarding changes in ML trunk lean, the default medium effect size was selected. For an alpha level of 0.05 and power of 80%, the estimated sample size was n = 24.

### Protocol

Participants were assessed for symptom severity (i.e. Hoehn & Yahr and MDS-UPDRS part III), cognition (MMSE), vision (Bailey-Lovie High Contrast visual acuity) and falls efficacy (FES-I). Following these assessments, reflective markers were placed on anatomical landmarks in accordance with the full-body Plug-in-Gait kinematic model (Vicon Nexus, Version 2.6, Oxford Metrics Ltd., Oxford, United Kingdom). Participants were barefoot and wore shorts and a crop top for women or no top for men.

Three-dimensional gait analysis was completed over 4 conditions across 2 sessions. Session 1 (Baseline, Intervention and Post-intervention conditions) lasted ~ 1.5 h, whilst Session 2 (Follow-up condition) lasted ~ 1 h and was completed 7 days (± 1 day) after Session 1. For each condition, across both sessions, 5 walking trials were recorded (including at least one trial with a clean foot strike on the force plate for the left foot and another for the right foot). Participants completed both assessments in an optimally medicated state (i.e. ON-phase).

Session 1 consisted of participants completing the Baseline condition walking trials, which involved walking at their own self-selected pace along an 8-m walkway. Following the Baseline condition feedback familiarization commenced; participants were asked to stand in the centre of the walkway, face the projection screen displaying biofeedback, and slowly move their trunk from left to right with increasing amplitude. Once participants had all their questions about the biofeedback tool and task answered to their satisfaction, they completed a minimum of three walking familiarization trials. The Intervention condition required participants to focus on the biofeedback displayed on a 2.16 m^2^ projector screen placed at the end of the walkway. Calculation and display of the biofeedback is described below. Following walking familiarization, 20 biofeedback Intervention trials were completed. Immediately following the Intervention trials, participants rested for 3-min, then completed 2-min of walking without feedback, to become refamiliarized with walking in the laboratory environment without visual biofeedback prior to data collection for the Post-intervention condition. Participants then completed the Post-intervention condition, where they walked without feedback.

For Session 2, participants returned to the laboratory to complete the Follow-up condition, where they walked without feedback along the walkway, again, adhering to the Baseline condition protocol.

### Data collection

During all walking trials, a 20-camera Vicon three-dimensional motion analysis system (Oxford Metrics Ltd., Oxford, United Kingdom) recorded gait kinematics at 150 Hz. This system was synchronized with a single ground-embedded force plate (1500 Hz; Advanced Mechanical Technology Inc., Watertown, MA, USA) located in the centre of the walkway to determine foot-strike and toe-off events.

### Biofeedback

**Fig. 1:**
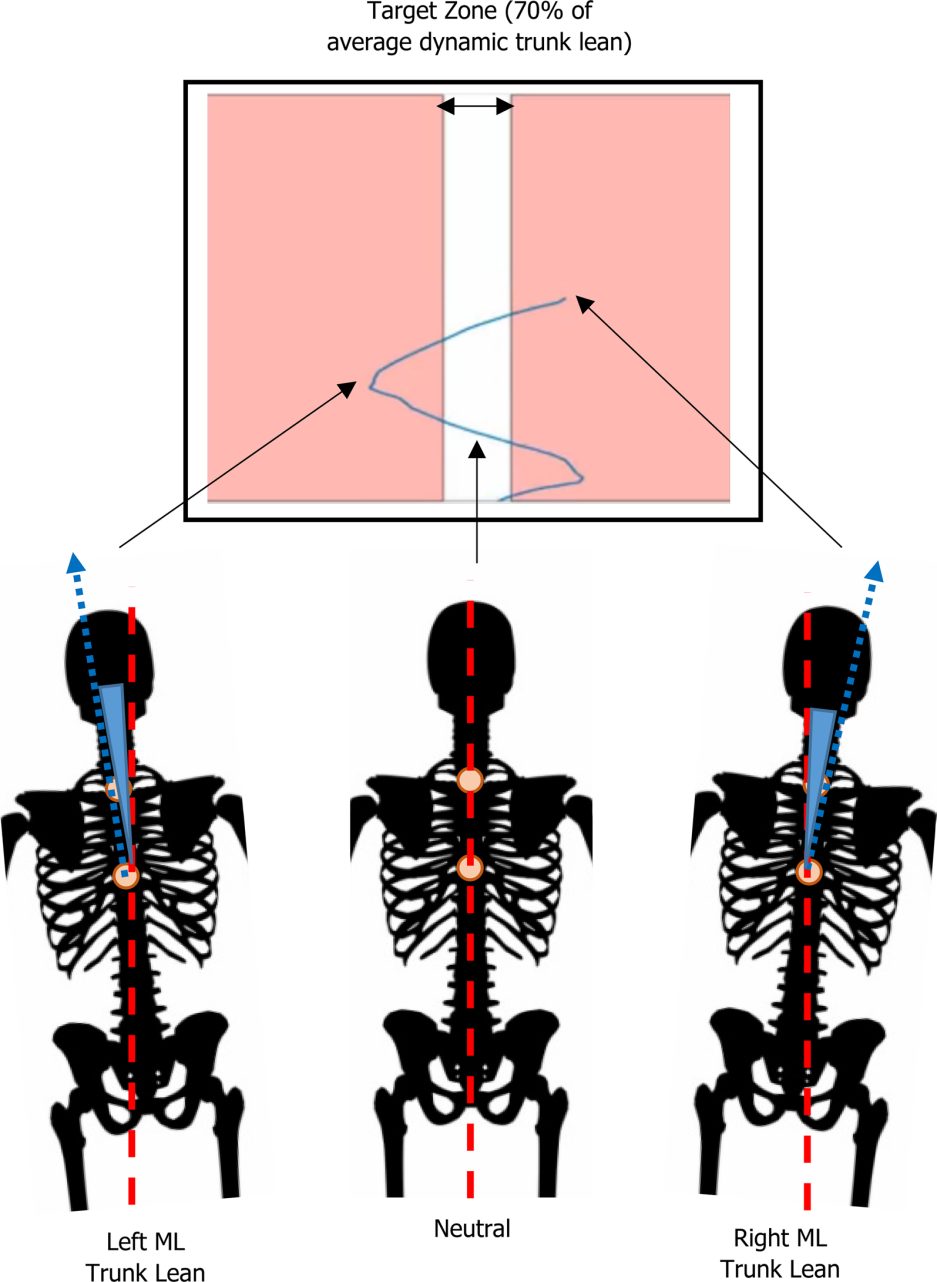
Representation of the visual projection of biofeedback during the Intervention walking condition. As the participants walked, their mediolateral trunk lean was calculated in real-time and the resultant angle (represented as a moving blue line) along with a target zone (the white space between the red shaded areas) was projected onto a screen (the black box at the top of the image) that was placed at the end of the walkway in front of the participant

Trunk marker trajectories were computed in real-time using Vicon Nexus software (Version 2.6, Vicon Motion Analysis, Oxford, England) and streamed to MATLAB (MathWorks, Massachusetts, USA), where a customized program calculated the ML lean of the trunk in the frontal plane of the laboratory. These data were projected onto a graph (Fig. 1) and displayed as a moving line. The horizontal axis representative of the magnitude of ML trunk lean (degrees) and the vertical axis representative of time (s). During the Intervention condition, participants were provided with a target reduction of 30% of their peak ML trunk lean relative to Baseline. Whilst other biofeedback studies have employed target changes of 50% or more in kinematics [13], given the retraining target in the current study was related to dynamic equilibrium in a population at risk of falling, the lesser target reduction of 30% was used. This desired modification was visually represented in real-time on the biofeedback projection by a centrally located white space (target zone), with neighboring red outer boundaries used to indicate motion beyond the target (Fig. 1).

### Data analysis

Three-dimensional reconstruction of marker trajectories was performed in Vicon Nexus. Marker trajectories were filtered using a generalized cross-validation quintic smoothing spline with a mean squared error of 15 mm^2^ [18]. Filtered trajectory data were then used to model segment kinematics and COM trajectories as well as spatiotemporal gait parameters (Vicon Plug-in-Gait). Trials were cropped to remove the first and last 2-m of each trial to ensure data represented constant velocity walking. Multiple steps in each of the five trials per condition (the final 5 trials of intervention condition), range of 10–24 strides were analyzed; foot-strike and toe-off events not corresponding with a clean force plate contact were identified from the vertical height of the lateral malleoli at foot-strike and toe-off during the force plate contact steps for that condition [19]. Peak ML trunk lean in the frontal plane of the laboratory was averaged across the movement from left to right and right to left peaks, and then averaged across trials. Absolute ML head, trunk and pelvis motion were measured by calculating the average range of motion of the mathematically-derived COM of each segment in the ML direction, relative to the plane of progression and reported in cm. Given the reported influence of gait velocity on ML motion of the axial skeleton [7, 8], consistent with these previous studies, absolute ML motion values were normalized to gait velocity (mean ML motion/gait velocity; reported in cm/m/s). Other variables evaluated because of their association with falls risk and dynamic equilibrium were step length [20], gait velocity [21–23] and COM to base of support distance (with positive values indicating the COM was positioned medial to the base of support) [8].

All statistical analyses were conducted using SPSS version 25 (IBM Corporation, New York, USA), with an alpha level set at 0.05. A repeated measures analysis of variance was used to compare variables across the four conditions (i.e. Baseline, Intervention, Post-Intervention, Follow-up). Data were checked for normality using the Shapiro–Wilk’s test and sphericity using Mauchly’s test. Where normality could not be assumed, data were log transformed and reassessed for normality prior to inferential statistics. Where assumptions of sphericity (*p* < 0.05) were violated, the Greenhouse–Geisser adjustment (ε) was utilized. For comparisons where significant main effects were present (*p* < 0.05), post-hoc comparisons were completed using the Tukey’s Least Significant Difference method. Standardised mean differences (SMD; Cohen’s *d*) were calculated as a measure of effect size.

## Results

Twenty-five individuals participated in Session 1, however one individual did not complete Session 2 due to travel issues (Additional file 1). Only participants who completed both Sessions (n = 24) were included in our analysis (Table 1).

**Table 1:** Participant demographics at baseline

	Participants (n = 24)
Demographics	
Age (years)*	68 (7.59)
Gender (male:female)	18:6
Height (cm)*	173 (7.08)
Mass (kg)*	83 (15.90)
BMI (kg/m^2^)*	28 (5.15)
Falls history and fear of falls	
Falls efficacy scale (/64)^#^	24 (8)
Fallers:Non-fallers^	12:12
Cognitive functioning	
Mini-mental state exam (/30)^#^	29.5 (2)
Disease severity	
H & Y Score (1:2:3)	6:16:2
MDS-UPDRS Score (/132)^#^	23 (10.75)

H&Y: Hoehn and Yahr. MDS-UPDRS: Movement Disorders Society—Unified Parkinson’s Disease Rating Scale

*Data are continuous and reported as Mean (Standard Deviation). Mean values are expressed to the nearest whole

^#^Data are scale based and reported as Median (Interquartile range). ^Fallers defined as having experienced a fall in the 12 months prior to data collection. H&Y: Hoehn and Yahr. MDS-UPDRS: Movement Disorders Society—Unified Parkinson’s Disease Rating Scale

^^^Fallers defined as having experienced a fall in the 12 months prior to data collection

### Gait biomechanics

All outcome measures were normally distributed, apart from normalized ML head, trunk and pelvis motion. These outcomes were log-transformed and reassessed for normality prior to further analysis. The primary variable of ML trunk lean was significantly different between conditions (F(3,69) = 9.22, p < 0.001). Post-hoc analysis show ML trunk lean decreased from Baseline to Intervention and Post-intervention conditions, with medium and small effect sizes (SMD = 0.5 and 0.32, respectively). However, no difference was observed between Baseline and Follow-up conditions (Table 2).

**Table 2:** Spatiotemporal and kinematic characteristics of participants during the four walking conditions

	Condition (mean (SD))						Effect size (SMD)					
	Baseline	Intervention	Post-Intervention	Follow-up		*Sig.*	B - I	B -PI	B -FU	I - PI	I -FU	PI -FU
Spatiotemporal characteristics												
Gait velocity (m/s)	1.12 (0.16)	1.01 (0.21)	1.10 (0.17)	1.12 (0.19)		a, b, d, e	0.67	0.27	–	0.50	0.67	–
Stride length (m)	1.22 (0.16)	1.13 (0.20)	1.20 (0.17)	1.22 (0.17)		a, b, d, e, f	0.56	0.25	–	0.37	0.58	0.29
Segmental motion												
ML Trunk lean (^o^)	4.19 (1.40)	3.48 (1.50)	3.88 (1.46)	4.04 (1.50)		a, b, d, e	0.50	0.32	–	0.39	0.57	–
Absolute ML Head motion (cm)	5.77 (1.39)	5.38 (2.07)	5.33 (1.23)	5.55 (1.49)		a, b	0.49	0.45	–	–	–	–
Absolute ML Trunk motion (cm)	5.01 (1.15)	5.04 (1.24)	4.81 (1.07)	4.85 (1.14)		ns	–	–	–	–	–	–
Absolute ML Pelvis motion (cm)	4.80 (0.89)	4.74 (0.91)	4.51 (0.79)	4.57 (0.74)		b, c, d	–	0.44	0.46	0.39	–	–
Normalised ML Head motion (cm/m/s)	5.38 (2.07)	5.71 (2.71)	5.07 (1.83)	5.22 (2.15)		ns	–	–	–	–	–	–
Normalised ML Trunk motion (cm/m/s)	4.67 (1.70)	5.41 (2.35)	4.57 (1.57)	4.57 (1.76)		a, d, e	0.50	–	–	0.46	0.51	–
Normalised ML Pelvis motion (cm/m/s)	4.45 (1.40)	5.03 (1.86)	4.27 (1.24)	4.26 (1.32)		a, d, e	0.54	-	–	0.52	0.57	–
COM to base of support distance (cm)*	4.69 (1.17)	4.37 (1.20)	4.51 (1.24)	4.48 (1.28)		a	0.34	–	–	–	–	–

“SD”, standard deviation. “SMD”, standardised mean difference. “ML”, mediolateral. “COM”, centre of mass. *Larger values indicate a more medial position of the COM relative to the base of support. “ns”, no difference between conditions. “a”, difference between baseline and intervention. “b”, difference between baseline and post-intervention. “c”, difference between baseline and follow-up. “d”, difference between Intervention and post-intervention. “e”, difference between intervention and follow-up. “f”, difference between post-intervention and follow-up. Sig: Statistically significant difference between conditions. “B”, Baseline. “I”, Intervention. “PI”, Post-intervention. “FU”, Follow-up

All statistically significant differences are to the *p* < 0.05 level. SMD values; Small = 0.2–0.5; Medium = 0.5–0.8; Large > 0.8

Consistent with the primary analysis, absolute ML head motion was less in the Intervention and Post-Intervention conditions when compared with Baseline (F(1.83,42.16) = 2.14, p = 0.001; Table 2). Absolute ML pelvis motion was less than Baseline at the Post-Intervention and Follow-up conditions (F(3, 69) = 4.49; p = 0.003), despite no difference between the Baseline and Intervention conditions.

Normalized ML motion data, i.e. divided by gait velocity, did not follow the same pattern as the absolute ML motion data, with no differences observed in normalized ML head motion. Normalized ML trunk (F(1.88, 43.13) = 7.87, *p* = 0.002) and pelvis (F(1.83, 42.05) = 8.57, *p* = 0.001) motion in fact increased from the Baseline to the Intervention condition with medium effect sizes (SMDs = 0.5 and 0.54, respectively). Both outcomes returned to levels similar to Baseline in the Post-Intervention and Follow-Up conditions.

Analysis of gait velocity (*p* < 0.001) and stride length (*p* = 0.001) indicated participants walked slower (F(1.74, 39.93) = 16.59) and took shorter strides (F(1.87, 43.17) = 18.77) during the Intervention condition compared with Baseline. These decreases were sustained during the Post-intervention condition, although the effect sizes were small (SMDs = 0.25 and 0.27, respectively) and both returned to baseline levels at Follow-up (Table 2). COM to base of support distance also decreased from the Baseline to Intervention conditions, indicating a less medial position of the COM relative to the base of support, with a small effect (F(2.49, 57.17) = 3.26; SMD = 0.34), but no other differences were observed.

## Discussion

This is the first study to examine whether it is possible to modify the normalized ML motion of the axial skeleton during walking gait in people with PD. Our findings support the primary hypothesis and demonstrate that people with PD can decrease ML trunk lean during gait with the assistance of visual biofeedback. The differences observed between the Baseline and Intervention conditions are in line with previous literature that found individuals with PD are able to utilize visual biofeedback to modify trunk position and lateral swaying motions during upright standing with [24] and without [16] an external perturbation. The secondary hypothesis was partially supported in that the decrease in absolute ML head motion during the intervention may be indicative of decreased falls risk, however increases in the relative ML trunk and pelvis motion, and a more lateral positioning of the COM relative to the base of support, during the intervention may be indicative of an increased risk of falls.

ML trunk lean was also reduced relative to Baseline in the Post-intervention condition. This provides evidence of short-term retention of the biofeedback-induced adaptations. No retention of effect was observed during the 1-week Follow-up condition however. These findings suggest that one intervention session was insufficient to allow for notable skill retention in individuals with PD. Whilst skill retention in a young healthy population is achievable following a single biofeedback session [25], it has been reported that repetition over several sessions (e.g. weeks or months) is required for people with PD [26, 27]. Future research may seek to determine whether longer lasting effects can be achieved with greater exposure to the biofeedback method over an extended time period.

Despite the efficacy of the intervention for reducing ML trunk lean, the provision of biofeedback also resulted in changes in kinematic and spatiotemporal outcomes that may be suggestive of poorer dynamic equilibrium and thus an increased likelihood of falls. Of note, gait velocity and stride length decreased during the Intervention condition relative to all other conditions. As gait velocity is the result of distance covered per unit of time, concurrent decreases in stride length were not surprising and may be suggestive of poorer dynamic equilibrium [4]. Decreased dynamic equilibrium was also reflected in a decreased COM to base of support distance during the Intervention condition, indicative of greater ML motion of the whole-body COM. Given the influence gait velocity has on several measures of dynamic equilibrium, the biomechanical consequences of biofeedback interventions on gait velocity and subsequent measures of dynamic equilibrium must be given further consideration.

Normalized ML trunk and pelvis motion (i.e. that divided by gait velocity) increased from the Baseline to Intervention condition, before decreasing again during the Post-intervention and Follow-up conditions. Contrary to this, there was no difference in absolute ML trunk and pelvis motion between Baseline and Intervention conditions. Thus, it appears likely that the observed increase in normalized ML trunk and pelvis motion during the Intervention condition occurred primarily as a result of the decrease in gait velocity during this condition. The reduction in gait velocity and subsequent effect upon normalized ML motion measures may be explained by the use of a cueing strategy that sought to provide individuals with greater spatial awareness. As PD individuals are often found to exhibit a decreased postural reserve (i.e. muscle strength, sensory motor integration and higher-level cortical control), increased attention is required to maintain posture and dynamic equilibrium. Re-weighting of attention to a separate motor or cognitive task diminishes the attention that can be assigned to controlling dynamic equilibrium and may result in inhibition of the automaticity of gait [28]. In conjunction, a deliberate reduction in gait velocity may have been utilized to allow greater time to adequately process the additional visual information prior to making a postural adjustment. Similar re-weighting of attentional resources has been noted in dual-tasking research where individuals often prioritize all elements of the task equally, rather than giving greatest importance to dynamic equilibrium [29, 30].

It is interesting to note that the increases in normalized ML trunk and pelvis motion were not accompanied by a significant change in normalized ML head motion. These findings may be indicative of successful dampening of motion by the pelvis and trunk, preventing unfavorable ML motion of the head [31, 32]. While both greater head and pelvis motion have been linked with increased falls risk in PD [4], they are strongly correlated with each other. Thus, it is not known which of these measures, or potentially both, are causative of the observed increased falls risk. Regardless, as there was no decrease in either of these measures with the intervention, an alternative approach should be considered in any attempt to translate the observed reduction in ML trunk lean to normalized ML head and pelvis motion. Future work might consider whether directly providing feedback regarding ML head and/or pelvis motion instead of 2D trunk lean, can better facilitate changes in these variables.

Visual biofeedback proved able to successfully decrease ML trunk lean, yet the influence of this approach upon falls risk is less clear given the observed changes in ML pelvis and trunk motion. A revised biofeedback intervention approach may therefore be necessary to achieve the desired gait modification, with the consideration of biofeedback effect on gait velocity of importance. Use of a treadmill would allow gait velocity to be controlled across conditions, potentially removing the influence of gait velocity changes on normalized ML motion. This form of intervention would allow for greater exposure to the biofeedback, which may enhance the potential effects of the intervention. The use of treadmill-based interventions is supported by prior literature, which shows it can successfully modify step length and gait velocity [33, 34].

There are limitations to this study that must be acknowledged. First, it was beyond the scope of this research to restrict individuals to meet specific gait impairments. Individuals with greater baseline ML motion may have had greater scope to decrease ML motion, potentially impacting the observed responses to the biofeedback. Nevertheless, given the known association between greater ML motion and falls risk, any decrease to ML motion has the potential to benefit all individuals with PD, particularly considering the high incidence of falls in this population. Second, the biofeedback employed a target reduction in trunk lean of 30%. This is less than biofeedback studies in otherwise healthy populations that targeted reductions of 50 and 80% [13, 25]. As the current study was targeting a movement pattern associated with falls in a population at high risk of falling, based on pilot investigations, we considered the 30% target to represent a significant challenge for participant’s without unduly elevating falls risk. It should be noted that a larger target reduction may have elicited a greater change in our outcome variables. Third, despite findings suggesting it was feasible to modify absolute ML head motion through changes to ML trunk lean, resultant changes to falls risk cannot be assumed without prospective falls analysis. The current study serves to inform future prospective research by reporting the immediate and short-term effects of the intervention on biomechanical factors associated with falls risk in PD.

## Conclusions

In conclusion, ML trunk lean was found to decrease with the provision of real-time biofeedback but returned to baseline levels following cessation of biofeedback. Despite observed decreases in ML trunk lean, normalized ML trunk and pelvis motion increased due to the intervention. This potential decrease in dynamic equilibrium may be explained by the reduction in gait velocity observed in response to an elevated attentional demand. These findings suggest that while the biofeedback approach used in this study may be useful for effecting short-term changes in trunk lean for people with PD, the potential negative effect upon other gait outcomes must be further evaluated. Additional research is needed to better understand how biofeedback can be used as a potential tool for biomechanical alteration within the PD population.

## Supplementary Information

**Additional file 1.** Participant flow through the study.

## Data Availability

The dataset used during the current study are available from the corresponding author on reasonable request.

## Abbreviations

PD: Parkinson’s disease
COM: Centre of mass
ML: Mediolateral
MMSE: Mini mental state exam
SMD: Standardised mean difference
