# Supplementary material for: Gait biofeedback training in people with Parkinson’s disease: a pilot study
Source: J Neuroeng Rehabil. 2022 Jul 16;19:72. doi: 10.1186/s12984-022-01051-1 (PMC9287978; doi:10.1186/s12984-022-01051-1)
Supplement: Supplementary file 1 — Additional file 1. Participant flow through the study. [file 12984_2022_1051_MOESM1_ESM.docx]

**Additional file 1.** Participant flow through the study.

Session 2 completed

(n = 24)

Expressed an interest in participation

(n = 78)

Phone Screening

(n = 43)

Face-to-face screening

(n = 26)

Session 1 completed

(n = 25)

DBS (n = 5)

≥80 years old (n = 3)

Requires assistance walking (n = 2)

Other forms of Parkinsonism (n = 2)

Travel restrictions (n = 6)

No further contact made by individual (n = 17)

DBS (n = 2)

≥80 years old (n = 1)

Requires assistance walking (n = 2)

Recent Injury (n= 3)

=

Other forms of Parkinsonism (n = 1)

Unable to complete Session 2 (n = 1)

**Withdrawals:**

Passed: declined to participate further (n = 9)

Session 2 completed and Data analysed

(n = 24)
